# Dietary supplementation of polysaccharides from *Millettia speciosa* Champ. ex Benth on growth performance, immune function, antioxidant abilities and intestinal health of Wenchang chickens

**DOI:** 10.1186/s12917-025-04934-2

**Published:** 2025-07-23

**Authors:** Yu-hang Liu, Jie Liu, Xin Feng, Quan-Wei Liu, Rui-ping Sun, Wei Wu, Kun Ouyang, Jing-li Yuan, Yan Zhang, Xiu-ping Wang, Gui-Ping Zhao, Li-Min Wei

**Affiliations:** 1 Sanya Institute, Hainan Academy of Agricultural Sciences (Hainan Experimental Animal Research Center), Sanya, 572025 China; 2 Present Address: Hainan Key Laboratory of Tropical Animal Breeding and Epidemic Research, Institute of Animal Husbandry& Veterinary Research, Hainan Academy of Agricultural Sciences, Haikou, 571100 China; 3 Present Address: School of Life Science and Engineering, Foshan University, Foshan, 528051 China; 4 Key Laboratory of WenChang Chicken Breeding and Feeding, Hainan (Tan Niu) Wenchang Chicken Co., Ltd, Haikou, 571100 China

**Keywords:** *Millettia speciosa* Champ. ex Benth polysaccharide, Growth performance, Immune function, Antioxidant ability, Intestinal health

## Abstract

This study aims to systematically evaluate the effects of *Millettia speciosa* Champ. ex Benth polysaccharides (MSCP) on broiler chickens through comprehensive assessment of growth performance parameters, immune system modulation, and intestinal microbial community dynamics. A total of 576 healthy 80-day-old Wenchang chickens were randomly assigned to six experimental groups in a completely randomized design. The control group (Control) received a basal diet, while the antibiotic-treated group was supplemented with 2 g/kg chlortetracycline hydrochloride (CTC) as a positive control. Four experimental groups were supplemented with different concentrations of MSCP: 400 mg/kg (MSCP_400_), 800 mg/kg (MSCP_800_), 1600 mg/kg (MSCP_1600_), and 3200 mg/kg (MSCP_3200_). The study employed a replicated pen design with 8 replicates per treatment group, each containing 12 birds. The results showed that dietary MSCP significantly increased the final body weight (*P* < 0.001) and average daily gain of chickens (*P* = 0.017), particularly at a dosage of 800 mg/kg. We also found that serum CAT level (*P* = 0.030) and GSH-Px level (*P* = 0.011) were significantly higher in the MSCP group compared to the CTC group. Adding MSCP to the feed raised serum IL-4 level (*P* = 0.016) and significantly increased IgA level (*P* = 0.047) at 1600 mg/kg. MSCP at 400 and 800 mg/kg significantly increased villus height in the duodenum, jejunum, and ileum (*P* < 0.001 for duodenum and jejunum; *P* = 0.040 for ileum). Moreover, MSCP also increased the relative abundance of beneficial bacteria *Synergistota*, *Parabacteroides*, *Megamonas* and *Faecalibacterium*, and increased the diversity of intestinal flora. To sum up, adding MSCP to feed had a positive impact on production performance, comparable to antibiotics, particularly at a dosage of 800 mg/kg. This suggests that MSCP is a promising, safe, and effective alternative to antibiotics in feed additives.

## Introduction

In the past few decades, antibiotic growth promoters (AGPs) have been widely used in animal breeding to promote growth and prevent diseases [1]. Injecting antibiotics into chickens in the chicken breeding industry can help them resist harmful bacteria, but it can also lead to issues like flora imbalance and damage to the intestinal barrier [2]. Prolonged use of antibiotics in poultry production can create antibiotic residues and drug-resistant bacteria in products, posing a risk to human health [3]. Several countries have prohibited the use of AGPs in poultry feed, making it essential to develop safe, residue-free feed additives to enhance poultry health and immunity [4].

The chicken's intestinal tract is both the largest immune and digestive organ, crucial for absorbing nutrients and defending against pathogens [5]. The health of chickens is directly impacted by the condition of their intestines. The maintenance of intestinal morphology, the balance of intestinal flora, and the integrity of the intestinal barrier are essential factors in protecting against pathogen infiltration and ensuring overall intestinal health. Polysaccharides from plants have become important in the use of phytogenic feed additives (PFAs) in recent years. Previous studies have shown that plant polysaccharides can act as probiotics, promoting intestinal health and barrier function. Zhang et al*.* found that using crude Tieguanyin oolong tea polysaccharides (CTPS) significantly improved inflammatory bowel diseases (IBDs) in mice induced by dextran sulfate sodium salt (DSS) [6]. Li and others supplemented *Laminaria japonica* polysaccharide (LJPS) in the meat duck diet, which increased the abundance of beneficial bacteria in the intestines of meat ducks [7]. Xie et al*. *[8] discovered that *Ginseng* polysaccharides improved intestinal villus height and histomorphology in Xuefeng black bone chickens. Liu et al*. *[9] discovered that dietary algae-derived polysaccharides (ADP) increased occludin and ZO-1 expression in the duodenum of broilers, improving intestinal barrier function. These studies demonstrated that natural plant polysaccharides benefit the intestinal flora, intestinal barrier and intestinal tissue morphology of animals.

*Millettia speciosa* Champ. ex Benth is the root of *Liriodendron* in the Leguminosae family. It is found in various provinces in China, including Hainan, Fujian, Guangxi, Guangdong, Hubei, and Hunan. It is one of the"four southern medicines"in South China and plays a crucial role in chicken breeding by maintaining intestinal flora and homeostasis despite external factors and oxidative stress at cellular level. Animal antioxidant system can remove excess free radicals and prevent oxidative stress. Polysaccharides from *Millettia speciosa* (MSCP) have been shown to have antioxidant, anti-inflammatory, hypolipidemic, and antibacterial properties [10, 11]. Zhao et al*.* [12] discovered that MSCP has strong antioxidant properties by effectively scavenging OH- and 2,2-Diphenyl-1-picrylhydrazyl (DPPH) free radicals.

Wenchang Chickens in Hainan, China are known for their small head, shin, and claws, as well as short neck and legs. They have excellent meat qualities and temperature tolerance and more [13, 14]. Hainan is located in South China, with a tropical marine monsoon climate and hot summer weather, which also brings great challenges to the feeding and management of broilers. Adverse feeding and management can easily lead to oxidative stress in broilers. At present, MSCP is not applicable to broiler production. This experiment included chlortetracycline hydrochloride as a positive control for antibiotic in feed and introduced MSCP to assess its impact on immune function, antioxidant capacity, and intestinal health, exploring its potential as an antibiotic alternative. The study aims to show the benefits of MSCP as a safe, eco-friendly feed additive in poultry farming.

## Materials and methods

### Test material

The experimental chicken were procured from Hainan (*Tanniu*) Wenchang Chicken Co., Ltd. (Haikou, China). Chlortetracycline hydrochloride, HY-B1327, Med Chem Express. MSCP, with 50% purity, was sourced from Xian QuanAo Biotech Co., Ltd. in Shanxi, China.

### Animal experiment

A total of 576 healthy 80-day-old Wenchang chickens were randomly assigned to six experimental groups in a completely randomized design. The control group (Control) received a basal diet, while the antibiotic-treated group (CTC) was supplemented with 2 g/kg chlortetracycline hydrochloride as a positive control. Four experimental groups were supplemented with different concentrations of MSCP: 400 mg/kg (MSCP_400_), 800 mg/kg (MSCP_800_), 1600 mg/kg (MSCP_1600_), and 3200 mg/kg (MSCP_3200_). The study employed a replicated pen design with 8 replicates per treatment group, each containing 12 birds. For diet, according to the standard of"Nutrition requirement of Yellow Feather Broiler"(NY/T3645-2020), corn-soybean meal feed was prepared. The composition and nutrition level of the experimental diet are shown in Table 1. The feeding experiment was conducted at Yongfa Base, part of the Hainan Academy of Agricultural Sciences. The twelve experimental chickens are raised in a cage with access to feed and water, and exposed to a mix of artificial and natural light. Immunization carried out according to the routine immunization procedure. The trial lasts 40 days. The experimental birds were euthanized by injecting pentobarbital sodium (150 mg/kg) into the vein, following the 2020 AVMA Guidelines, and then weighed. All the experimental procedures applied in this study were reviewed and approved by the Experimental Animal Ethics Committee of Animal Husbandry and Veterinary Research Institute, Hainan Academy of Agricultural Sciences (HNSYY20230604, Approval date: 4 June 2023).

**Table 1:** Composition and nutrient level of diets (dry matter basis, %)

Ingredients	Control
Corn	72.1
Bran	4.5
Soybean meal	17.1
Soybean oil	3.0
CaHPO_4_	1.0
Limestone	1.0
Premix^a^	1.0
NaCl	0.3
Analyzed nutrient content	
ME, kcal/kg	3062.05
Crude protein	14.50
Crude Fat	6.04
Crude fiber	2.44
Calcium	0.69
Phosphorous	0.51
Available phosphorus	0.38
Lysine	0.65
Methionine	0.39
Arginine	0.87

^a^The premix provided the following per kg of diets per kg diet: Cu 10 mg, Fe 80 mg, Mn 60 mg, Zn 70 mg, I 2 mg, Se 0.40 mg, Vitamin A 10000 IU, Vitamin E 10 mg, Vitamin B2 12 mg, Vitamin B6 23 mg, Vitamin B12 63.50 mg, nicotinic acid 15 mg, folic acid 0.50 mg, pantothenic acid 10 mg, biotin 0.15 mg, cyanocobalamin 10 μg. Metabolizable energy (ME) is calculated and the rest is measured

### Analysis of nutritional composition of feed

The crude fat and crude fiber contents of the feed and fecal samples were determined according to the methods of National Standards of the People's Republic of China GB/T 6433–2006 (China National Standard, 2006), GB/T 6434–2022 (China National Standard, 2022) respectively. The total phosphorus content in the feed was determined by spectrophotometry. The calcium content in feed was determined by EDTA disodium complexometric titration (GB/T 6436–2018). The crude protein content in feed was determined by the Kjeldahl method (GB/T 6432–2018). The crude fat content in feed was determined by The contents of lysine, methionine and threonine in the feed were determined by the conventional acid hydrolysis method (GB/T 18246–2019).

### Growth performance

The feed consumption, initial body weight (IBW) and final body weight (FBW) of the chickens during the experiment were recorded in each repetition, and the average daily feed intake (ADFI), average daily gain (ADG) and feed conversion rate (FCR) were calculated. The formula are as follows:

$$\text{ADG}=(\text{FBW}-\text{IBW})/\text{Trial days}$$

$$\text{ADFI}=\text{Total feed intake}/\text{Trial days}$$

$$\text{FCR}=\text{ADFI}/\text{ADG}$$

### Serum biochemical indicators

At the end of the experiment, one chicken from each pen was chosen at random, weighed, and euthanized by bleeding. Blood was taken from the pterygoid vein and put into an EDTA tube for centrifugation at 2500 g for 15 min. The supernatant was then extracted and stored at −80 °C to test serum biochemical levels. Serum biochemical parameters, such as levels of immunoglobulin A (IgA), IgM, IgG, interleukin-4 (IL4), IL-10, tumor necrosis factor-a (TNF-a), TNF-β, total antioxidant capacity (T-AOC), GSH-Px, catalase (CAT), and malondialdehyde (MDA), were all determined by using kit. The kit from Nanjing Jiancheng Institute of Biological Engineering was used according to the manufacturer's instructions.

### Intestinal morphology

The duodenum, jejunum, and ileum were cut into 1 cm segments, fix in 4% paraformaldehyde, and stain with hematoxylin and eosin. The villus height and crypt depth were measured using a fluorescence microscope to calculate the VH/CD ratio.

### Determination of intestinal barrier-related factors by mRNA

RNA is extracted from the jejunum with the RNA Easy Fast Total RNA Kit (TIANGEN, China) [15], and its concentration and purity were measured using an IMPLEN P330 ultra-micro spectrophotometer. Total RNA was reverse transcribed into cDNA using a TIANGEN kit and stored at −20 °C. The qPCR system undergoes a 5-min pre-denaturation at 95 °C, followed by 40 cycles of 95 °C for 30 s, 60 °C for 30 s, and 72 °C for 15 s. β-actin serves as the internal reference gene, and gene expression levels were quantified using the 2^−ΔΔCT^ method. Primer sequences were provided by Beijing Tsingke Biotech Co., Ltd., as detailed in Table 2.

**Table 2:** Primer sequences

Gene	Primer sequences (5′ → 3′)	Product length	TM ℃
*β-actin*	F: CATTGTCCACCGCAAATGCT R: AAGCCATGCCAATCTCGTCT	109	57.2
*Occludin*	F: TGCTTTTGCCCAAGCAGGAA R: TGTGGGAGAGGCACCAGTTG	145	60.0
*Claudin-1*	F: GGTATGGCAACAGAGTGGCT R: CAGCCAATGAAGAGGGCTGA	91	60.0
*MUC2*	F: TTCATGATGCCTGCTCTTGTG R: CCTGAGCCTTGGTACATTCTTGT	93	58.0
*TLR-4*	F: TGGATCTTTCAAGGTGCCACA R: AGTGTCCGATGGGTAGGTCA	198	57.0

### Intestinal microflora analysis

Genomic DNA from cecal stool samples was extracted using a Tiangen kit, and its concentration and purity were assessed via 1% agarose gel electrophoresis. The V3-V4 regions of the 16S rRNA gene were amplified using primers 515 F and 806R. PCR products were purified with magnetic beads, quantified, and mixed. They were then analyzed with 2% agarose gel electrophoresis and recovered using a Qiagen kit. Library construction was done with the NEB Next® UltraTM II FS DNA kit and quantified using Qubit and Q-PCR. Once quantified, PE 250 sequencing was conducted on a NovaSeq 6000.

The QIIME toolkit filters original sequences for quality, assigning high-quality reads to the same OTU with ≥ 97% similarity using the Uplink algorithm. Species diversity and complexity were analyzed with QIIME (V1.9.1), and PCoA was conducted. Microbial functions predicted by PICRUSt at level 3 (LDA > 2) were analyzed using the LEfSe algorithm.

### Statistical analysis

The study used a completely randomized design and processed preliminary data with SPSS 17. Results are shown as mean ± SD (in tables) or mean ± SEM (in figures). A T-test compared a single variable between two groups, while one-way ANOVA with Tukey's post hoc test and linear and quadratic regression analyzed interactions among multiple variables. A *P*-value of ≤ 0.05 indicated significance.

## Results

### Growth performance

As shown in Table 3, FBW in the CTC, MSCP_400_, MSCP_800_, MSCP_1600_ and MSCP_3200_ groups were significantly higher than that in the control group (*P* < 0.001). ADG level was also significantly higher than in the control group (*P* = 0.038). No significant differences were observed in feed weight ratio (*P* = 0.854) and average daily feed intake (*P* = 0.898) among groups.

**Table 3:** Effects of MSCP on growth performance of Wenchang Chickens

Items^1^	Treatment^2^						*p-*value		
	Control	CTC	MSCP_400_	MSCP_800_	MSCP_1600_	MSCP_3200_	*P*_ANOVA_	*P*_Linear_	*P*_Quadratic_
IBW (g)	1260.63 ± 1.13	1260.00 ± 1.34	1261.25 ± 1.57	1260.00 ± 1.34	1261.25 ± 1.57	1262.50 ± 1.34	0.799	0.312	0.465
FBW (g)	1723.16 ± 16.09^b^	1844.01 ± 7.51^a^	1801.25 ± 12.05^a^	1801.66 ± 15.29^a^	1790.92 ± 18.20^a^	1792.84 ± 22.60^a^	< 0.001	0.163	0.003
ADG (g)	15.15 ± 0.58^a^	16.69 ± 0.35^a^	16.72 ± 0.13^a^	16.26 ± 0.2`1^a^	16.43 ± 0.20^a^	16.41 ± 0.23^a^	0.017	0.071	0.020
ADFI (g)	72.80 ± 2.48	76.13 ± 1.13	73.28 ± 1.13	72.26 ± 1.13	73.44 ± 1.13	74.09 ± 1.13	0.898	0.895	0.903
FCR	5.91 ± 0.28	5.87 ± 0.56	5.42 ± 0.56	5.96 ± 0.23	5.56 ± 0.22	5.86 ± 0.23	0.805	0.825	0.533

Different superscript letters indicate significant difference (*P* < 0.05). Data are presented as the mean ± SD. The number of samples in each group is 8

^1^*IBW* Initial body weight, *FBW* Final body weight, *ADG* Average daily gain, *ADFI* Average daily feed intake, *FCR* Feed conversion ratio

^2^*CTC* Chlortetracycline hydrochloride, *MSCP Millettia speciosa* Champ. ex Benth polysaccharides

### Immune index

Table 4 showed that serum IgA levels were significantly higher in the MSCP_1600_ group compared to the Control group (*P* = 0.047). IL-4 levels were also significantly higher in the MSCP group compared to the Control group (*P* = 0.016) but were not significantly different from the CTC group (*P* > 0.05).

**Table 4:** Effects of MSCP on immune index of Wenchang Chickens

Items	Treatment^1^						*p-*value		
	Control	CTC	MSCP_400_	MSCP_800_	MSCP_1600_	MSCP_3200_	*P*_ANOVA_	*P*_Linear_	*P*_Quadratic_
IgA mg/ml	1.57 ± 0.27^b^	1.61 ± 0.27^b^	1.63 ± 0.16^b^	2.40 ± 0.72^ab^	3.19 ± 0.54^a^	2.11 ± 0.11^ab^	0.047	0.032	0.535
IgM mg/ml	29.32 ± 0.64	29.86 ± 0.28	29.36 ± 0.40	29.67 ± 0.61	29.69 ± 0.21	31.39 ± 0.27	0.232	0.034	0.116
IgG mg/ml	11.48 ± 0.63	12.60 ± 1.22	11.78 ± 0.28	15.28 ± 1.93	14.84 ± 0.92	11.23 ± 1.31	0.056	0.411	0.066
TNF-α ng/L	146.58 ± 21.20	196.25 ± 37.06	162.15 ± 25.84	146.72 ± 21.81	148.20 ± 10.90	141.36 ± 24.99	0.645	0.346	0.517
TNF-β ng/L	20.32 ± 1.76	17.56 ± 2.17	24.24 ± 2.42	25.95 ± 4.84	25.88 ± 2.93	22.33 ± 1.47	0.250	0.196	0.241
IL-4 ng/L	48.31 ± 1.21^b^	52.60 ± 1.14^ab^	56.72 ± 1.37^a^	56.48 ± 2.25^a^	57.32 ± 1.57^a^	57.13 ± 1.39^a^	0.016	< 0.001	0.016
IL-10 ng/L	125.72 ± 12.28	133.67 ± 17.48	116.11 ± 16.17	102.09 ± 17.99	147.17 ± 25.34	128.24 ± 18.02	0.639	0.806	0.509

Different superscript letters indicate significant difference (*P* < 0.05). Data are presented as the mean ± SD. The number of samples in each group is 8

^1^*CTC* Chlortetracycline hydrochloride, *MSCP Millettia speciosa* Champ. ex Benth polysaccharides

### Antioxidant abilities

Table 5 showed that GSH-Px levels were significantly higher in the MSCP group compared to the CTC group (*P* = 0.017) but were not significantly different from the Control group (*P* > 0.05). CAT levels were significantly higher in the MSCP group compared to the Control group (*P* = 0.030) but were not significantly different from the CTC group (*P* > 0.05).

**Table 5:** Effects of MSCP on antioxidant abilities of Wenchang Chickens

Items	Treatment^1^						*p-*value		
	Control	CTC	MSCP_400_	MSCP_800_	MSCP_1600_	MSCP_3200_	*P*_ANOVA_	*P*_Linear_	*P*_Quadratic_
T-AOC mM	0.37 ± 0.10	0.36 ± 0.06	0.43 ± 0.05	0.50 ± 0.06	0.38 ± 0.07	0.40 ± 0.06	0.666	0.616	0.351
MDA nmol/mg	5.23 ± 0.90	6.26 ± 1.07	6.83 ± 0.80	5.56 ± 0.57	6.48 ± 0.60	5.99 ± 0.33	0.682	0.609	0.373
GSH-Px U/g	165.53 ± 1.78^a^	152.31 ± 9.74^b^	169.82 ± 1.49^a^	170.11 ± 2.13^a^	164.71 ± 2.42^a^	171.92 ± 1.75^a^	0.011	0.037	0.746
CAT U/mL	6.34 ± 1.27^b^	19.40 ± 5.63^a^	14.39 ± 1.03^a^	15.53 ± 0.59^a^	17.55 ± 1.65^a^	15.85 ± 2.12^a^	0.030	0.057	0.090

Different superscript letters indicate significant difference (*P* < 0.05). Data are presented as the mean ± SD. The number of samples in each group is 8

^1^*CTC* Chlortetracycline hydrochloride, *MSCP* *Millettia speciosa* Champ. ex Benth polysaccharides

### Intestinal morphology

**Fig. 1:**
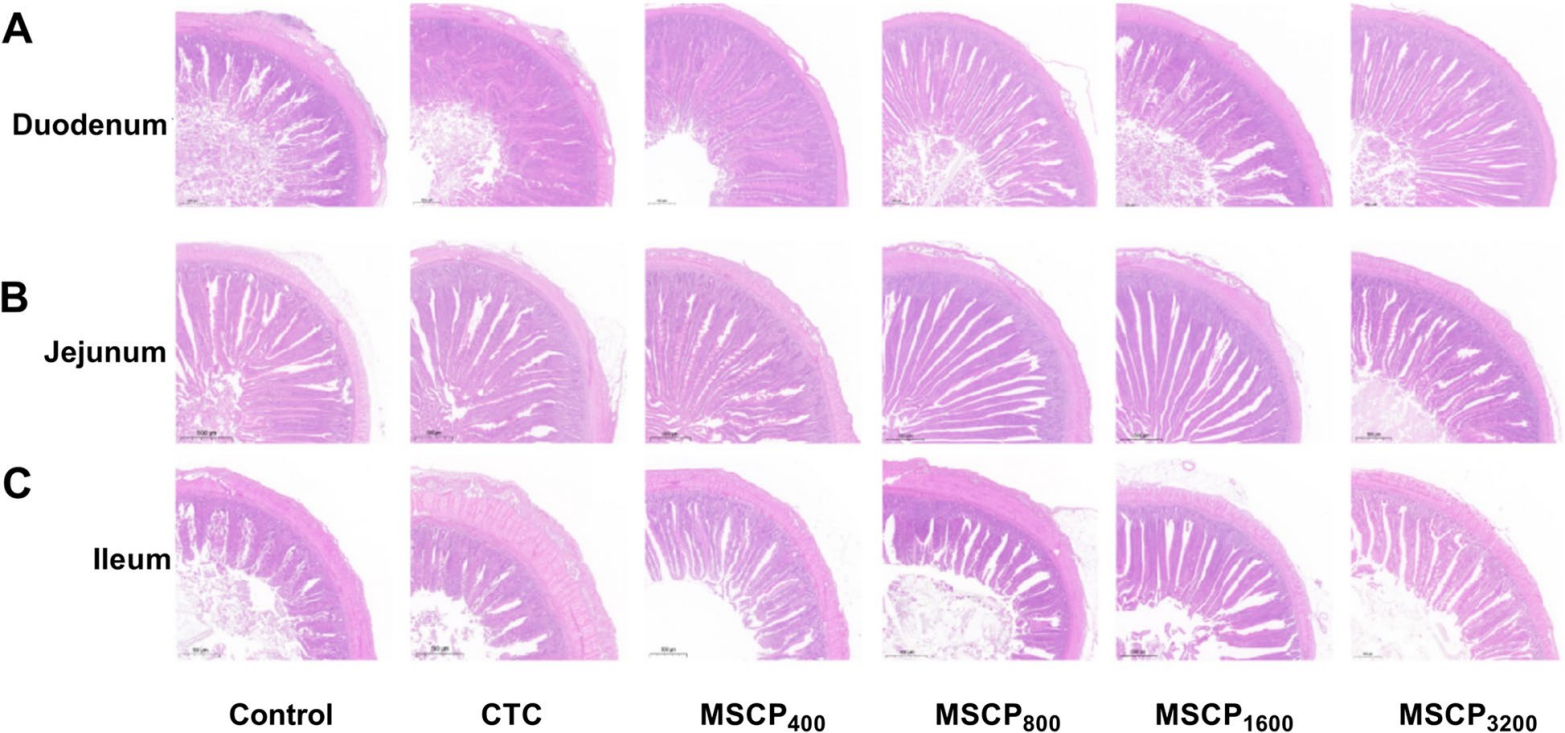
Intestinal histomorphology. Histomorphology of duodenum (**A**), jejunum (**B**), ileum (**C**). Scale bars: 100 µm

**Fig. 2:**
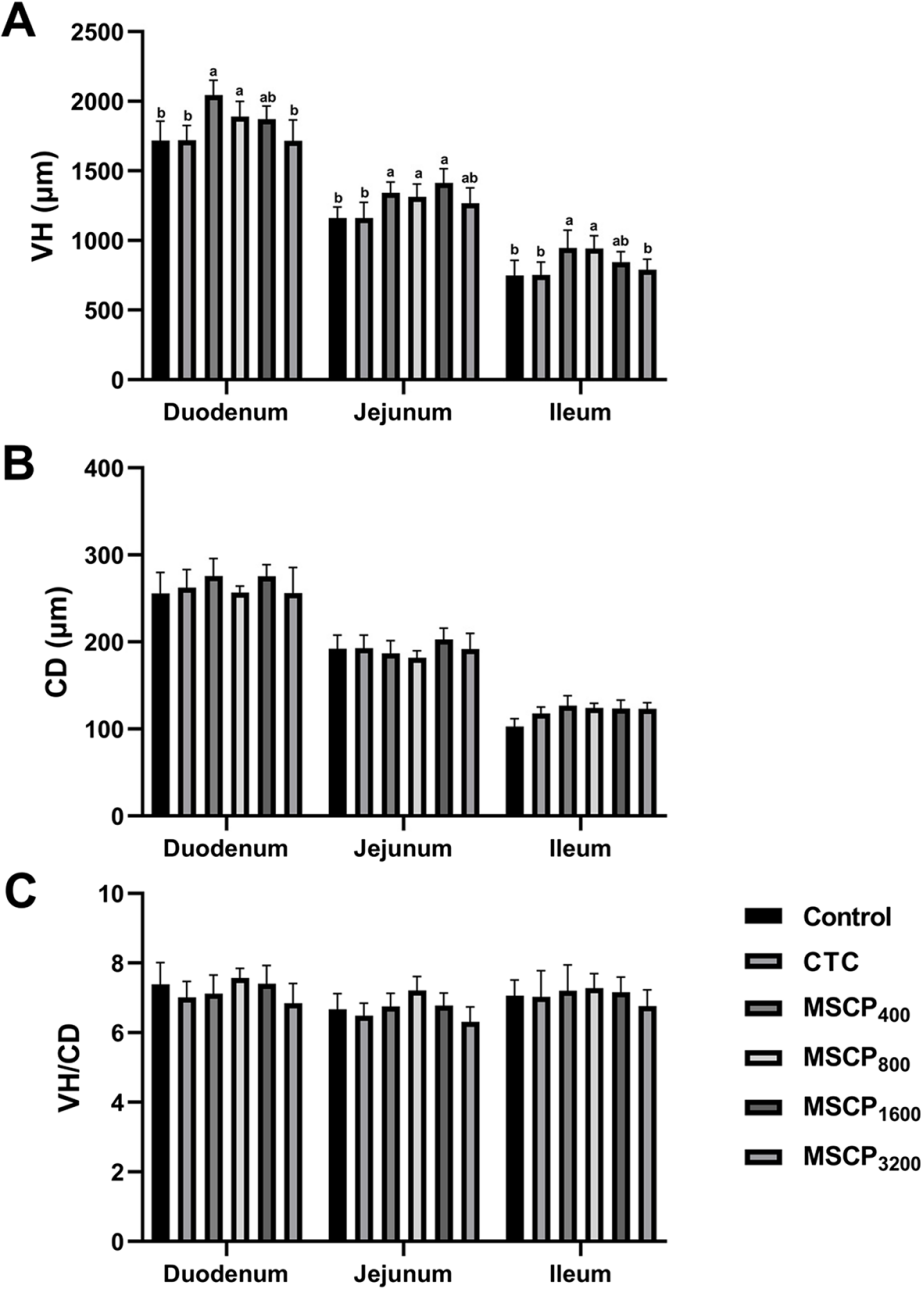
Morphologic analysis of intestinal histomorphology. **A** VH. **B** CD. **C** VH/CD. Different superscript letters indicate significant difference (*P* < 0.05). Data are presented as the mean ± SEM. The number of samples in each group is 3

In Figs. 1 and 2, the villus height in the duodenum of the MSCP_400_ and MSCP_800_ groups were significantly higher than in the Control, CTC, and MSCP_3200_ groups (*P* < 0.001) but were not significantly different from the MSCP_1600_ group (*P* > 0.05). The villus height in the jejunum of the MSCP_400_, MSCP_800_, and MSCP_1600_ groups was significantly higher than in the Control and CTC groups (*P* < 0.001) but was not significantly different from the MSCP_3200_ group (*P* > 0.05). The height of ileum villi in the MSCP_400_ and MSCP_800_ groups was significantly higher than in the Control, CTC, and MSCP_3200_ groups (*P* < 0.001). MSPC groups exhibited denser and more complete intestinal villi morphology compared to the Control and CTC groups, as seen in Fig. 1.

### Sequencing overview

**Fig. 3:**
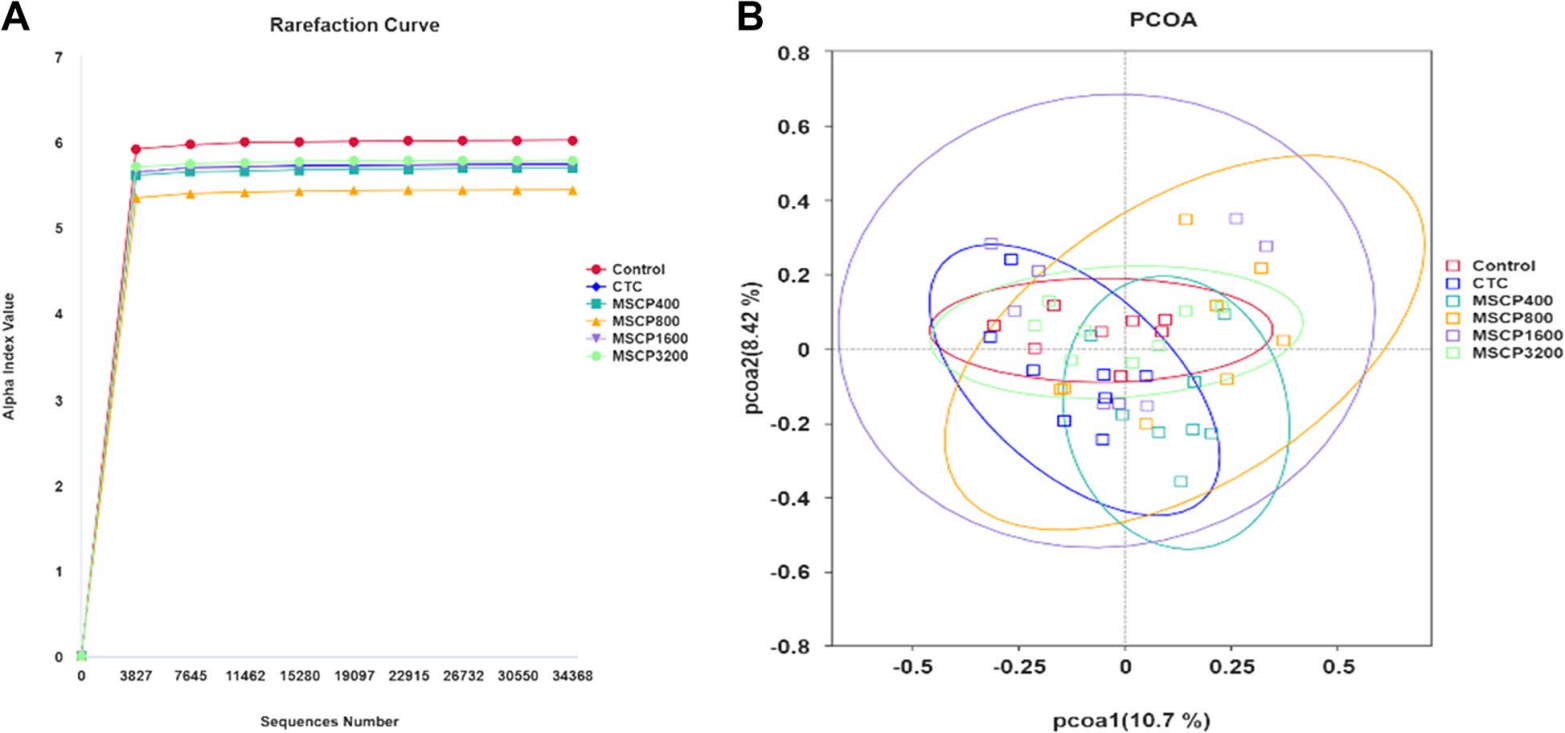
Shannon rarefaction curves and principal coordinate analysis (PCoA) of cecal bacterial communities. **A** shannon rarefaction curves of OUTs clustered. **B** PCoA of bacterial communities of chickens using Bray-Curti’s distance. The number of samples in each group is 8

In Fig. 3A, Shannon rarefaction curves stabilized, with an estimated Goods coverage of over 99.5% per sample, indicating nearly complete microbial community coverage. Figure 3B showed that the first and second principal components explained 10.7% and 8.42% of the sample variances, respectively, with some overlap between groups. Groups were not completely separated. As shown in Table 6, dietary MSCP had no significant effect on alpha-diversity indices of the bacterial communities (*P* > 0.05).

**Table 6:** Alpha diversity index of cecal microorganisms

Items	Treatment^1^						*p-*value		
	Control	CTC	MSCP_400_	MSCP_800_	MSCP_1600_	MSCP_3200_	*P*_ANOVA_	*P*_Linear_	*P*_Quadratic_
Chao1	584.96 ± 23.42	550.67 ± 34.98	588.04 ± 30.84	599.13 ± 25.84	575.76 ± 21.86	558.46 ± 17.01	0.766	0.836	0.520
Observed_otus	571.01 ± 24.67	540.00 ± 33.17	568.43 ± 35.69	603.86 ± 25.60	563.96 ± 20.71	562.30 ± 18.48	0.717	0.783	0.622
Shannon	5.79 ± 0.15	5.74 ± 0.26	5.78 ± 0.12	5.80 ± 0.12	5.83 ± 0.05	5.94 ± 0.14	0.971	0.464	0.615
Simpson	0.94 ± 0.01	0.93 ± 0.02	0.95 ± 0.01	0.92 ± 0.02	0.94 ± 0.01	0.95 ± 0.01	0.452	0.632	0.249
Goods coverage	0.99 ± 0.00	0.99 ± 0.00	0.99 ± 0.00	0.99 ± 0.00	0.99 ± 0.00	0.99 ± 0.00	0.813	0.215	0.786

Data are presented as the mean ± SD. The number of samples in each group is 8

^1^*CTC* Chlortetracycline hydrochloride, *MSCP Millettia speciosa* Champ. ex Benth polysaccharides. Different superscript letters indicate significant difference (*P* < 0.05)

### Relative abundances of phylum and genus

**Fig. 4:**
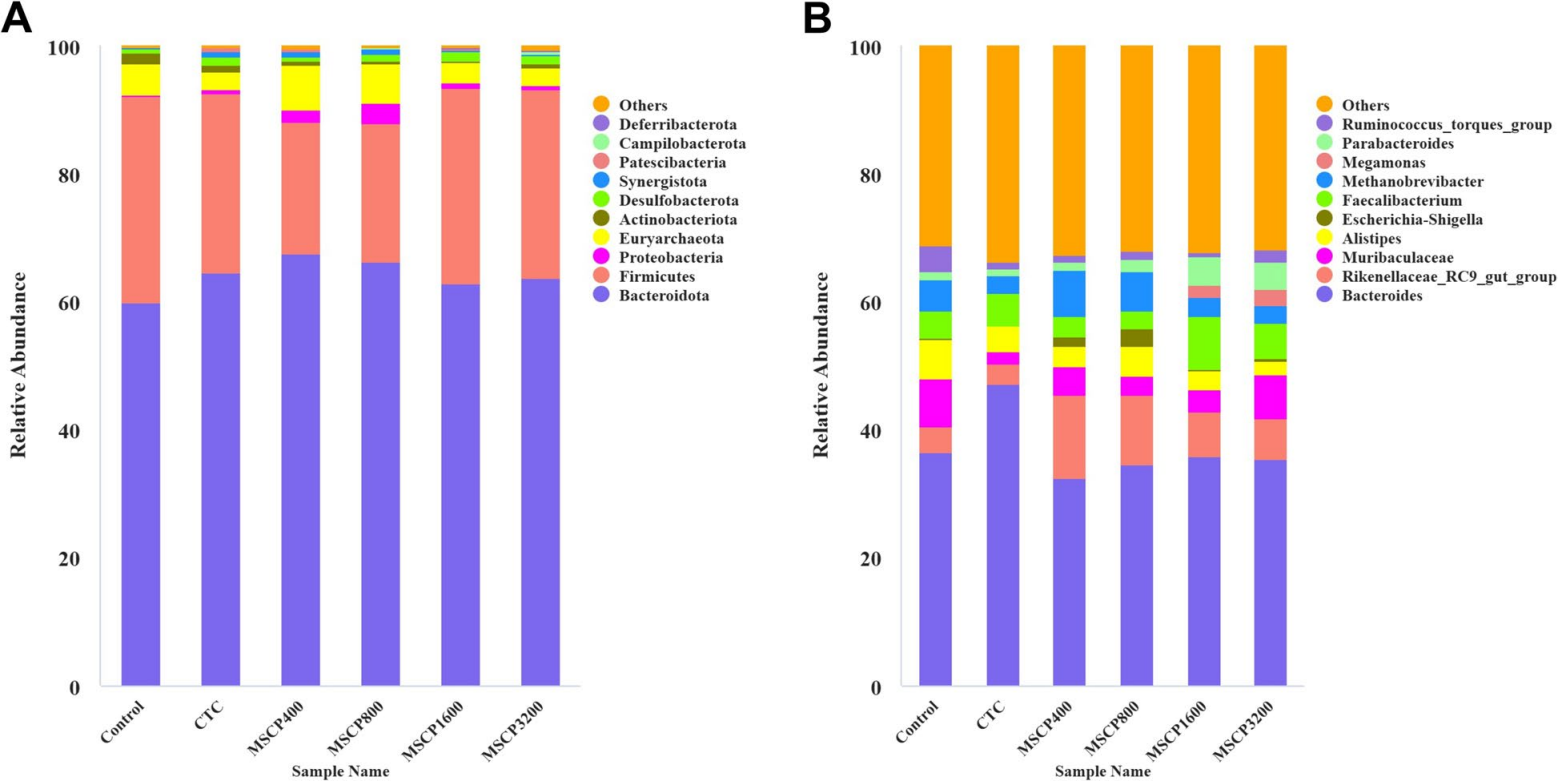
Relative abundance (%) of key microbial communities in chicken cecum at the phylum (**A**) and genus level (**B**). The number of samples in each group is 8

**Fig. 5:**
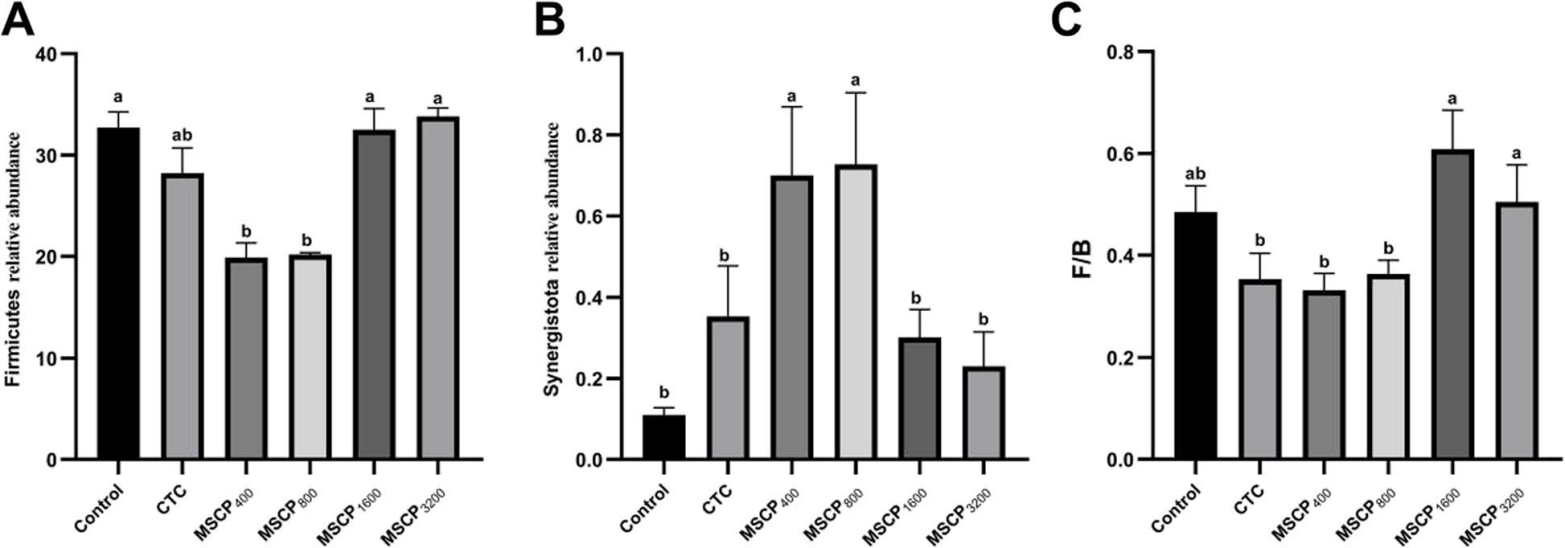
Phylum-level abundance differences in chicken cecum flora. **A** *Firmicutes*. **B** Synergistota. **C** *Firmicutes* to *Bacteroidota* ratio. Different superscript letters indicate significant difference (*P* < 0.05). Data are presented as the mean ± SEM. The number of samples in each group is 8

Figures 4A and 5A showed that *Bacteroidota* and *Firmicutes* were the most abundant phyla in each group, constituting 95% of the total abundance. The Firmicutes abundance in the MSCP_400_ and MSCP_800_ groups was significantly lower than in other groups (*P* = 0.02). *Synergistota* was more abundant in the MSCP_400_ and MSCP_800_ groups compared to the Control, MSCP_1600_, and MSCP_3200_ groups (Fig. 4B) (*P* = 0.03). The *Firmicutes* to *Bacteroidota* ratio in the MSCP_400_, MSCP_800_, and CTC groups was significantly lower than in the MSCP_1600_ and MSCP_3200_ groups (*P* = 0.013) but was not significantly different from the Control group (Fig. 4C) (*P* > 0.05).

**Fig. 6:**
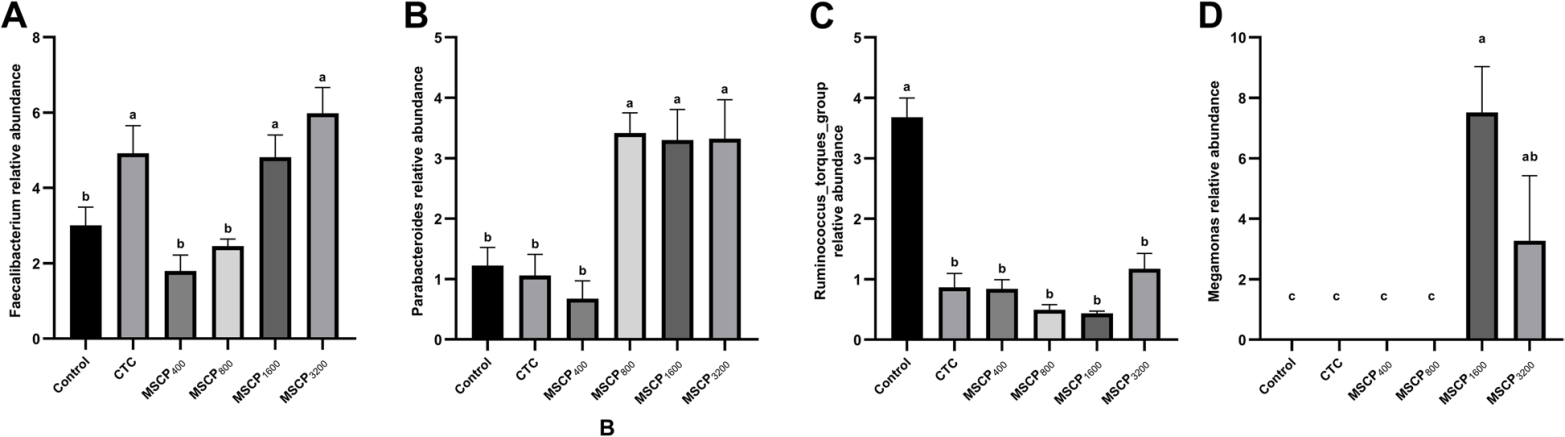
Genus-level abundance differences in chicken cecum flora. **A** *Faecalibacterium*. **B** *Parabacteroides*. **C** *Ruminococcus_torques_group*. **D** *Megamonas*. Different superscript letters indicate significant difference (*P* < 0.05). Data are presented as the mean ± SEM. The number of samples in each group is 8

**Fig. 7:**
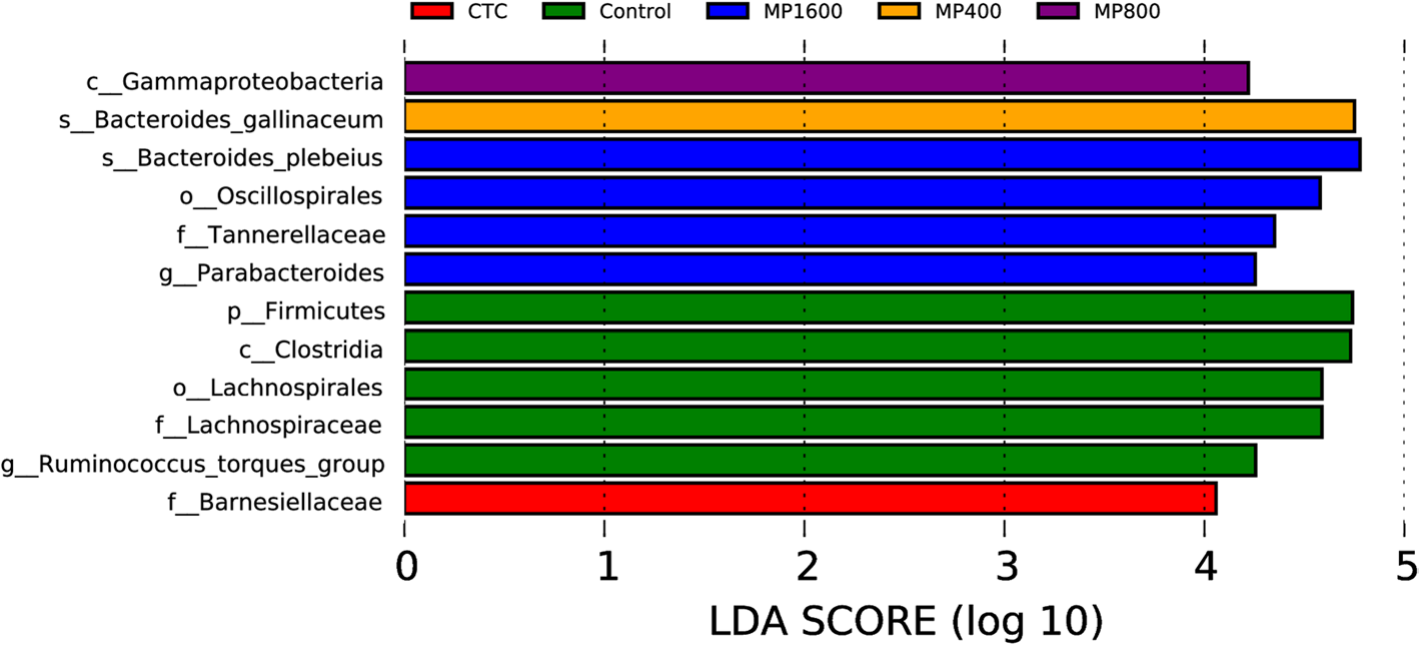
Histogram of LDA value distribution (LDA score is greater than 4)

The most common genera in the study were *Bacteroides* and *Rikenellaceae_RC9_gut_group* (Figs. 4B and 6). *Faecalibacterium* was significantly more abundant in the MSCP_1600_ and MSCP_3200_ groups compared to the Control, MSCP_400_, and MSCP_800_ groups (*P* < 0.001) but was not significantly different from the CTC group (Fig. 6A). *Parabacteroides* abundance significantly increased with higher levels of MSCP addition (Fig. 6B) (*P* < 0.001). The *Ruminococcus_torques_group* was significantly more abundant in the control group compared to other groups (Fig. 6C) (*P* = 0.001). *Megamonas* were only detected in the MSCP_1600_ and MSCP_3200_ groups, as seen in Fig. 7.

### LEfSe analysis

LEfSe (linear discriminant analysis; LDA > 4) analysis compared multiple groups to identify species with significant differences in abundance. LefSe analysis was used to identify biomarker groups within the dataset. Figure 7 displays the main species influencing group distinctions. Twelve dominant flora were identified, including 5 groups in the Control group (*Firmicutes, Clostridia, Lachnospirales, Lachnospiraceae, and Ruminococcus_torques_group*), 1 group in the CTC group (*Barnesiellaceae*), 1 group in the MSCP_400_ group (*Bacteroides_gallinaceum*), 1 group in the MSCP_800_ group (*Gammaproteobacteria*), and 4 groups in the MSCP_1600_ group (*Bacteroides_plebeius, Oscillospirales, Tannerellaceae, and Parabacteroides*) (Fig. 7).

### mRNA expression in jejunum

**Fig. 8:**
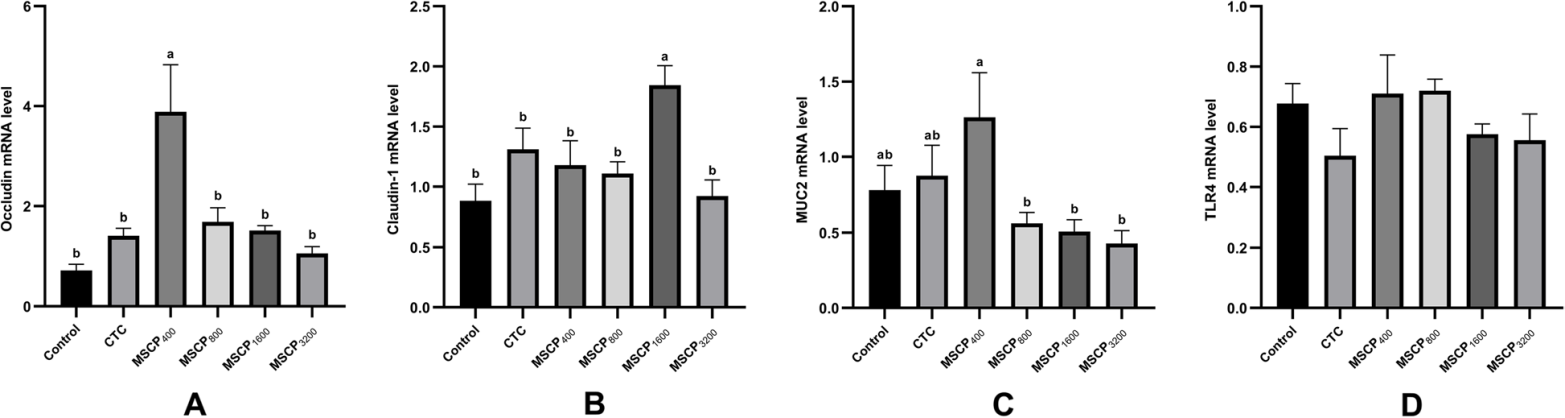
Jejunum mRNA expression of genes related to intestinal barrier function. **A** *Occludin*. **B** *Claudin-1*. **C** *MUC2*. **D** *TLR4*. Different superscript letters indicate significant difference (*P* < 0.05). Data are presented as the mean ± SEM. The number of samples in each group is 8

In the jejunum, Occludin mRNA levels in the MSCP_400_ group were significantly higher than in other groups (Fig. 8A) (*P* = 0.003). Claudin-1 mRNA levels in the MSCP_1600_ group were significantly higher than those in other groups (Fig. 8B) (*P* = 0.014). MUC-2 mRNA levels in the MSCP_400_ group were significantly higher than in the MSCP_800_, MSCP_1600_, and MSCP_3200_ groups (Fig. 8C) (*P* = 0.0014) but were not significantly different from the Control and CTC groups (*P* > 0.05). There was no notable variation in TLR4 mRNA levels among the groups (*P* > 0.05).

## Discussion

Overuse of antibiotics in animals has led to the rapid growth of drug-resistant bacteria and harmed the environment. The poultry industry focuses on chicken intestinal health to improve growth performance. In this study, we proved that dietary supplementation with different doses of MSCP significantly improved growth performance, enhanced immune and antioxidant function, and improved intestinal morphology and intestinal health.

The impact of adding dietary MSCP to broilers has not been previously studied. In the study, MSCP increased FBW and ADG significantly, but did not have a significant effect on ADFI and FCR. The best improvement was seen with the addition of 800 mg/kg of MSCP. This aligns with previous findings that plant polysaccharides can enhance the growth performance of broilers [16]. Prior research has demonstrated that including algae-derived polysaccharides (ADPs) in broiler diets enhances growth performance and increases villus height in the duodenum, jejunum, and ileum [17]. In this study, MSCP improved broiler VH to different extents and enhanced intestinal morphology compared to the control group and CTC group. An increase in VH is thought to improve nutrient absorption in the intestines [18, 19]. Based on the results, feeding broilers MSCP may enhance their intestinal structure. More research is needed to fully understand the positive effects of MSCP on growth performance and its potential mechanism.

Serum immunoglobulins are essential for the humoral immune system. During an infection, the body's immunoglobulin levels rise to combat pathogens and antigens, helping to neutralize viruses and dissolve bacteria, indicating the body's immune function. Adding 1600 mg/kg MSCP greatly raised IgA and IgG levels. Inflammatory cytokines are crucial in the immune response. IL-4 is a key cytokine that helps bone marrow cells become dendritic cells, boosting antigen processing and antibody production [20]. In this study, MSCP also notably raised serum IL-4 levels. Adding MSCP to broiler diet may boosts immune function by activating lymphocytes, stimulating proliferation and differentiation, and increasing secretion of immunoglobulins and cytokines.

Maintaining a balance between oxidation and antioxidation is crucial for cell physiology, energy metabolism, and overall bodily functions [21]. Excessive reactive oxygen species (ROS) from oxidative stress can harm animal metabolism, damage cell structure, speed up oxidation, and lead to diseases [22, 23]. The balance of free radicals in animals requires antioxidant enzymes like SOD, GSH-Px, and CAT [24]. In this study, we discovered that serum CAT and GSH-Px levels were significantly higher in the MSCP group compared to the CTC group. The results of the present study demonstrate that the natural antioxidant polysaccharide MSCP from plants has potential for improving broiler production.

A healthy intestine is crucial for nutrient absorption and preventing harmful microorganisms in animals, particularly in broiler production. Longer villi in the intestinal tract promote better nutrient absorption, aiding in the growth and development of broilers. Numerous studies have shown that natural plant polysaccharides can improve the morphology of intestinal villi in broiler chickens [8, 25]. In this study, adding MSCP improved the shape of chicken intestinal villi shape compared to the Control and CTC groups. The jejunum is the longest segment in a chicken's small intestine and plays a key role in nutrient absorption. Its barrier stability is crucial for the overall intestinal health of broilers. Occludin, claudin-1, and MUC2 are essential proteins for maintaining intestinal barrier integrity and permeability. A study found that MSCP improved intestinal health in mice with CTX-induced enteritis by increasing the expression of genes related to mucosal integrity and enhancing intestinal morphology [11]. We measured the essential gene expression in the jejunum and found that Occludin and MUC2 mRNA levels were significantly higher in the MSCP_400_ group compared to other groups, while Claudin-1 mRNA levels were significantly higher in the MSCP_1600_ group. The research suggests that adding MSCP to feed may enhance intestinal health by increasing villus height and regulating intestinal barrier gene expression.

Intestinal microorganisms are linked to intestinal structure, immune barrier function, and inflammation [5]. Maintaining a healthy gut microbiome is essential for animal growth and development, affecting intestinal tissue development and nutrient absorption [26, 27].

In this study, there was a significant decrease in *Firmicutes* abundance at the *phylum* level in the MSCP_400_ and MSCP_800_ groups compared to the Control group. *Firmicutes* play a role in breaking down protein, fat, and other complex organic substances in the body, indicating the level of obesity in animals [28].

Excessive fat deposition in hen's liver may cause fatty liver and lead to a decline in production performance. Reducing *F/B* (*Firmicutes*/*Bacteroidota*) can enhance host energy metabolism [29]. *Synergistota* is more abundant in the MSCP_400_ and MSCP_800_ groups compared to the Control, MSCP_1600_, and MSCP_3200_ groups. *Synergistota*, including various genera like *anaerobic Corynebacterium* and *Clostridium*, is found throughout the environment and plays a beneficial role in maintaining intestinal balance in animals [30]. *Faecalibacterium* can reduce intestinal inflammation by producing butyric acid, inducing anti-inflammatory factor IL-10, and increasing tight protein expression to reduce intestinal permeability, benefiting broiler health [31]. Some studies have shown that *Faecalibacterium* and *Parabacteroides* can work together to regulate intestinal T cells and produce butyric acid, which has immunomodulatory and anti-inflammatory effects [32]. In this study, *Faecalibacterium* is more abundant in MSCP_1600_ and MSCP_3200_ groups compared to the control, MSCP_400_, and MSCP_800_ groups, with its abundance increasing as MSCP is added. *Parabacteroides* also increased in MSCP groups, suggesting immunomodulatory and anti-inflammatory effects of MSCP. Additionally, only the MSCP_1600_ and MSCP_3200_ groups detected *Megamonas*. *Megamonas* ferments carbohydrates to produce SCFA, promoting intestinal health [33]. Furthermore, LEfSe analysis is used to estimate the relative abundance of intestinal microflora in each group. LEfSe analysis also showed that the dominant species in MSCP group are significantly different from those in control group and CTC group, which indicated that the abundance of intestinal microflora in chickens fed with MSCP is changed. Therefore, we speculate that MSCP improves intestinal function mainly by increasing the abundance of beneficial bacteria, reducing the abundance of harmful bacteria and increasing the diversity of intestinal flora to maintain intestinal health.

## Conclusions

In conclusion, the addition of MSCP to the feed significantly improved the growth performance of *Wenchang* chicken, which may be attributed to the improvement of intestinal morphology and the increase in the abundance of beneficial bacteria in the intestinal tract. MSCP can boost immunity in chickens by increasing levels of IgA, IgG, and IL-4 in serum, as well as enhancing antioxidant capacity by raising levels of CAT and GSH-Px. In this experiment, adding MSCP to the feed had a positive impact on production performance, comparable to antibiotics, particularly at a dosage of 800 mg/kg. This suggests that MSCP is a promising, and safe in feed additives. Future efforts should concentrate on investigating MSCP's active ingredients, understanding its mechanism, and assessing its long-term safety to establish its potential as a feed antibiotic substitute.

## Data Availability

The original contributions generated for this study are included in the article. Further inquiries can be directed to the corresponding author.

## Abbreviations

ADFI: Average daily feed intake
ADG: Average daily gain
ADPs: Algae-derived polysaccharides
AGPs: Antibiotic growth promoters
CAT: Catalase
CD: Crypt depth
CTC: Chlortetracycline hydrochloride
CTPS: Crude Tieguanyin oolong tea polysaccharides
DPPH: 2,2-Diphenyl-1-picrylhydrazyl
DSS: Dextran sulfate sodium salt
F/B: *Firmicutes* / *Bacteroides*
FBW: Final body weight
FCR: Feed conversion rate
GSH-Px: Glutathione peroxidase
IgA: Immunoglobulin A
IgG: Immunoglobulin G
IgM: Immunoglobulin M
IL-4: Interleukin-4
IL-10: Interleukin-10
LJPS: *Laminaria **japonica* Polysaccharide
MDA: Malondialdehyde
MSCP: *Millettia speciosa* Champ. ex Benth
Muc-2: Mucin-2
PFAs: Phytogenic feed additives
ROS: Reactive oxygen species
SOD: Superoxide dismutase
T-AOC: Total antioxidant capacity
TNF-α: Tumor necrosis factor-α
TNF-β: Tumor necrosis factor-β
VH: Villus height
VH/CD: Villus height/crypt depth
